# Addison's Disease Presenting as Acute Renal Failure and Hyperkalemic Paralysis: A Rare Presentation

**DOI:** 10.1155/2021/3103011

**Published:** 2021-12-22

**Authors:** Kundan Jana, Kalyana Janga, Sheldon Greenberg, Amit Gulati

**Affiliations:** Division of Nephrology and Hypertension, Maimonides Medical Center, Brooklyn, NY 11219, USA

## Abstract

Hyperkalemic paralysis in the setting of acute renal failure can lead to a missed or delayed diagnosis of adrenal insufficiency as the raised potassium can be attributed to the renal failure. Acute kidney injury as the presenting manifestation in an adrenal crisis due to Addison's disease has been rarely reported in the literature. Here, we present the case of a young 37-year-old male who came with hyperkalemic paralysis and acute renal failure needing emergent hemodialysis. He had no past medical history and no medication history. His hyponatremia, hypotension, and hyperkalemia pointed to a picture of adrenal insufficiency confirmed by undetectable serum cortisol, elevated ACTH, renin, and low aldosterone levels and imaging. Replacement steroid therapy was given, and the patient made a steady recovery. He was advised on the importance of compliance to treatment at discharge to prevent another crisis event. Acute renal failure with hyperkalemia as a presenting manifestation of Addison's disease can be very misleading. It is especially important to be vigilant of adrenal insufficiency in such patients as the hyperkalemia is resistant to standard therapy of insulin dextrose and can precipitate fatal arrhythmia if treatment is delayed.

## 1. Introduction

Primary adrenal insufficiency is a rare disorder with low incidence and is usually a challenging diagnosis to make due to the vagueness of the symptoms patients present with. Patients with chronic Addison's disease present with weakness, fatigue, anorexia, skin hyperpigmentation, or acute adrenal crisis. Commonly encountered electrolyte abnormalities include hyponatremia and hyperkalemia. Hyperkalemic paralysis with acute renal failure can be a very misleading presentation of Addison's disease as the life-threatening hyperkalemia can be misdiagnosed as a consequence of the kidney injury. Very few cases in the literature have so far been reported where acute renal failure was the presenting manifestation of primary adrenal insufficiency [1–3]. Here, we present such a case of a young 37-year-old male who came in with acute flaccid paralysis secondary to life-threatening hyperkalemia and acute renal failure.

## 2. Case Report

**Figure 1:**
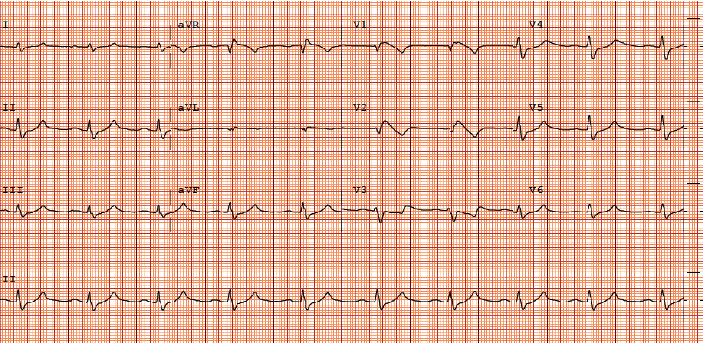
Pre-cardiac arrest ECG.

A 37-year-old presented with acute weakness and inability to move his limbs after waking up from sleep. He experienced similar symptoms of weakness in the past after a prolonged period of rest, but none of them were as severe. There was no history of recent medication or drug use and no history of diarrhea, infections, or trauma. He had no other significant past medical history. On arrival to the Emergency Department, the patient was hypotensive with a blood pressure of 98/50 and a heart rate of 84 per minute. The patient endorsed some improvement in weakness on the way to the emergency department (ED). However, physical examination was still significant for weakness in all four limbs with intact sensations and no fecal or urinary incontinence. The patient also complained of inability to walk. While taking history and drawing his labs, the patient went into cardiac arrest. ECG showed sinus rhythm with PR interval prolongation and QRS widening (Figure 1). He was intubated and resuscitated with return of spontaneous circulation after 6 minutes. His blood work taken prior to the cardiac arrest was significant for acute hyperkalemia of 8.8 mmol/l (normal 3.5–5 mmol/l), hyponatremia of 124 mmol/l (normal 135–145 mmol/l), and acute kidney injury with serum creatinine of 1.7 mg/dl (normal 0.5–1.3 mg/dl) (Table 1).

The patient underwent emergent renal replacement therapy (RRT) for life-threatening hyperkalemia and was transferred subsequently to the intensive care unit. To exclude CNS causes of paralysis, brain and cervical spine MRI were done and they were unremarkable. Viral causes were excluded with nasopharyngeal swab for the respiratory pathogen panel, including enteroviruses, and serology for HIV and CMV. Thyroid dysfunction, as a cause for paralysis, was excluded with normal serum thyroid stimulating hormone (TSH) and negative thyroid peroxidase antibodies. Urine tox screen was also negative. Autoimmune serology for vasculitis including ANCA, ANA, rheumatoid factor, anti-SSA, anti-SSB, anti-SCL-70, and double-stranded DNA were all negative. Obstructive uropathy as a cause of the hyperkalemia and acute renal failure was ruled out with a draining Foley catheter, and imaging showed no signs of retention. Normal creatinine phosphokinase (CPK) and myoglobin levels in pre-cardiac arrest labs ruled out rhabdomyolysis as a cause of the acute kidney injury (AKI). Hyponatremia, hyperkalemia, and hypotension pointed to a differential diagnosis of probable adrenal insufficiency. Accordingly, serum cortisol levels, ACTH levels, plasma renin activity, and aldosterone levels were obtained. Our patient had undetectable cortisol levels of <0.4 µg/dl, elevated ACTH level, elevated renin level, and low aldosterone levels (Table 1). The trans-tubular potassium gradient (TTKG) was calculated to be less than 5. A diagnosis of primary adrenal insufficiency was made and further confirmed by imaging which showed bilateral atrophic adrenal glands. Further workup revealed the presence of adrenal antibodies suggestive of autoimmune adrenal insufficiency. The patient was diagnosed with Addison's disease presenting in adrenal crisis.

**Figure 2:**
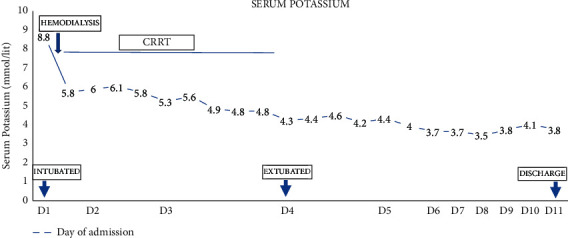
Course of hospitalisation with serum potassium levels.

After the first emergent session of hemodialysis, the patient remained in oligoanuric renal failure with hemodynamic instability and underwent continuous renal replacement therapy (CRRT) over the next 4 days. His potassium levels gradually came back to normal levels on CRRT (Figure 2). He made a steady recovery and was off RRT and extubated on the fourth day of hospitalisation. By this time, his weakness had completely resolved. The patient was finally discharged on maintenance of hydrocortisone 10 mg twice daily and fludrocortisone 0.1 mg/day with instructions for stress-dose hydrocortisone three times the usual dose in case of febrile illness and use of emergency injectable vial of hydrocortisone in case of vomiting and inability to take pills.

## 3. Discussion

Addison's disease is a rare endocrinological disorder with an estimated prevalence of 35–140 cases per million and incidence of 4 per million [4]. Tuberculosis (TB) was the most common cause of Addison's disease. However, with the advent of effective medication for TB, autoimmune causes are currently considered to be the most common etiology [3]. Adrenal insufficiency does not manifest until 90% of the gland has been destroyed or atrophied as seen in imaging. It is notoriously difficult to diagnose with vague and varied presentations. A study showed that up to 60% of patients were needed to be evaluated by at least 2 physicians with a median duration of diagnosis of up to 2 years, attesting to the insidious nature of the disease [5]. Presenting signs and symptoms of Addison's disease include weakness, fatigue, anorexia, and skin pigmentation with hypotension, hyponatremia, and hyperkalemia.

Acute adrenal crisis is a life-threatening presentation of Addison's disease seen in as many as 25% of the patients [3]. Acute renal failure as a presenting symptom is extremely rare with an incidence of 6% reported in the literature [3]. Oftentimes, such a presentation can lead to a missed or delayed diagnosis of adrenal insufficiency as the electrolyte disturbances of hyperkalemia and hyponatremia can be explained by the renal failure. Our patient presented with life-threatening hyperkalemia which could have been attributed to the acute renal failure. However, no discernable cause of the kidney injury provided a clue to another underlying disease pathology. Many pathophysiological mechanisms have been hypothesized on the cause for AKI in patients with adrenal insufficiency. Mineralocorticoid deficiency-induced intravascular volume depletion leading to decreased renal perfusion causing reduction in glomerular filtration rate was the likely cause of AKI in our patient [1].

Paralysis is a rare and reversible complication of hyperkalemia that can be categorized into primary and secondary forms. Primary hyperkalemic periodic paralysis is due to a congenital defect in the *SCN4A* gene affecting the sodium channel in muscles [6]. Secondary hyperkalemic paralysis can be due to acute or chronic renal failure, adrenal insufficiency, excessive ingestion of potassium, rhabdomyolysis, and various drugs like angiotensin converting enzyme inhibitors (ACEis), potassium-sparing diuretics, and cotrimoxazole, with renal failure being the most common cause [7, 8]. Hyperkalemic paralysis due to primary adrenal insufficiency is rare and can lead to fatal arrhythmias if not diagnosed early. A high index of suspicion should be maintained, especially in young patients, even when the picture is complicated by AKI, as seen in this patient.

Prompt institution of resuscitative and steroid replacement therapy is absolutely necessary in patients with acute adrenal crisis. Life-threatening hyperkalemia in such patients, as seen in our case, is resistant to the standard therapy of insulin dextrose. In fact, insulin dextrose can be detrimental in such patients for the danger of precipitating hypoglycemia as the patients are predisposed due to low glucocorticoid levels [1]. Renal replacement therapy was emergently administered in our patient to bring down the potassium levels and control fatal arrhythmias. Subsequently, the patient was stabilized and counselled upon discharge on the need for religious adherence to steroid therapy, the importance of stress-dose steroid replacement as warranted, and the need to carry a Medical Alert ID.

## 4. Conclusion

Acute adrenal crisis due to Addison's disease presenting as acute renal failure with hyperkalemic paralysis is extremely rare and can lead to delayed diagnosis. Physicians should include adrenal insufficiency in their differential in such cases as hyperkalemia in such patients is resistant to standard therapy and can precipitate arrhythmias if diagnosis is delayed.

## Figures and Tables

**Table 1:** Laboratory values.

Test	At admission	Day 3	Day 5	At discharge	6-month follow-up	Normal reference values
Glucose (mg/dl)	76	143	109	97	99	59–140
BUN (mg/dl)	32	25	28	23	14	7–21
Creatinine (mg/dl)	1.7	1.5	1.1	1	1.22	0.5–1.3
Serum sodium (mmol/l)	124	132	138	135	137	135–145
Serum potassium (mmol/l)	8.8	5.3	4	3.8	4.5	3.5–5
Serum chloride (mmol/l)	99	99	103	99	100	93–105
Serum CPK (IU/l)	280	1318				59–367
Serum osmolality (mosm/kg)	289					275–295
Renin (direct) (pg/ml)	>1500				47.1	<33
Plasma ACTH (pg/ml)	261		91.9			7.2–63.3
Serum cortisol (*μ*g/dl)	<0.4					6.7–27.6
Serum aldosterone (ng/dl)	2.8					<23
Serum TSH (mIU/l)	3.7					0.4–4
Urine osmolality (mosm/kg)	445			628		500–800
Urine sodium (mmol/l)	97			62		
Urine potassium (mmol/l)	19			50		

BUN: blood urea nitrogen; CPK: creatinine phosphokinase; units are given in parentheses.

## Data Availability

No data were used to support this study.
